# The prognostic impact of pre-treatment cachexia in resectional surgery for oesophagogastric cancer: a meta-analysis and meta-regression

**DOI:** 10.1093/bjs/znad239

**Published:** 2023-08-01

**Authors:** Leo R Brown, Judith Sayers, Michael S Yule, Thomas M Drake, Ross D Dolan, Donald C McMillan, Barry J A Laird, Stephen J Wigmore, Richard J E Skipworth

**Affiliations:** Clinical Surgery, University of Edinburgh, Royal Infirmary of Edinburgh, Edinburgh, UK; Clinical Surgery, University of Edinburgh, Royal Infirmary of Edinburgh, Edinburgh, UK; Clinical Surgery, University of Edinburgh, Royal Infirmary of Edinburgh, Edinburgh, UK; Centre for Medical Informatics, University of Edinburgh, Edinburgh, UK; Cancer Research UK Beatson Institute, Glasgow, UK; Academic Unit of Surgery, University of Glasgow, Glasgow Royal Infirmary, Glasgow, UK; Academic Unit of Surgery, University of Glasgow, Glasgow Royal Infirmary, Glasgow, UK; Institute of Genetics and Cancer, University of Edinburgh, Edinburgh, UK; Clinical Surgery, University of Edinburgh, Royal Infirmary of Edinburgh, Edinburgh, UK; Clinical Surgery, University of Edinburgh, Royal Infirmary of Edinburgh, Edinburgh, UK

## Abstract

**Background:**

Cancer cachexia is not purely an end-stage phenomenon and can influence the outcomes of patients with potentially curable disease. This review examines the effect of pre-treatment cachexia on overall survival, in patients undergoing surgical resection of oesophagogastric cancer.

**Methods:**

A systematic literature search of MEDLINE, EMBASE and Cochrane Library databases was conducted, from January 2000 to May 2022, to identify studies reporting the influence of cachexia on patients undergoing an oesophagogastric resection for cancer with curative intent. Meta-analyses of the primary (overall survival) and secondary (disease-free survival and postoperative mortality) outcomes were performed using random-effects modelling. Meta-regression was used to examine disease stage as a potential confounder.

**Results:**

Ten non-randomized studies, comprising 7186 patients, were eligible for inclusion. The prevalence of pre-treatment cachexia was 35 per cent (95 per cent c.i.: 24–47 per cent). Pooled adjusted hazard ratios showed that cachexia was adversely associated with overall survival (HR 1.46, 95 per cent c.i.: 1.31–1.60, *P* < 0.001). Meta-analysis of proportions identified decreased overall survival at 1-, 3- and 5-years in cachectic cohorts. Pre-treatment cachexia was not a predictor of disease-free survival and further data are required to establish its influence on postoperative mortality. The proportion of patients with stage III/IV disease was a significant moderator of between-study heterogeneity. Cachexia may have a greater influence on overall survival in studies where more patients have a locally advanced malignancy.

**Conclusion:**

Pre-treatment cachexia adversely influences overall survival following resection of an oesophagogastric malignancy.

## Teaser

Cancer cachexia is not purely an end-stage phenomenon and can even influence the outcomes of patients with potentially curable disease. In patients undergoing surgical resection of an oesophagogastric cancer, cachexia is adversely associated with overall survival (HR 1.46, 95 per cent c.i.: 1.31–1.60, *P* < 0.001). Consideration of cachexia, during the shared decision-making process, may improve risk stratification and facilitate targeted interventions.

## Introduction

Weight loss is a common presenting complaint for many patients who will go on to be diagnosed with cancer^1^. While its prevalence is highest among those with advanced stages of disease^2^, a significant proportion of patients with potentially curable cancers will also be affected, particularly in oesophagogastric (OG) cancer^3,4^. In such patient groups, a proportion of weight loss will often be attributable to direct mechanical or digestive issues caused by tumour burden; however, cachexia per se is also highly prevalent^5^. Cancer cachexia is ‘a multifactorial syndrome defined by an ongoing loss of skeletal muscle mass (with or without loss of fat mass)’^6^. It is driven by systemic inflammation, metabolic and endocrine dysfunction and leads to progressive functional impairment^7^. While there are a number of published definitions for cachexia^6,8,9^, with variations in their diagnostic criteria, involuntary weight loss is the central tenet of all.

A large body of literature has considered the association between radiologically evident sarcopenia and outcomes in patients with cancer^10^. Recent reviews have highlighted the high prevalence and adverse prognostic impact of sarcopenia in operable gastric^11^ and oesophageal cancers^12^. Sarcopenia is, however, often representative of the patient's pre-morbid body habitus^13^ and does not necessarily reflect dynamic disease-related wasting. Low muscle mass and density are endemic throughout a range of cancers sites and stages^14^ and are even frequently found in healthy individuals^15^. There is a comparative paucity of research examining cachexia in resectable OG cancer.

This review examined the influence of pre-treatment cachexia on overall survival in patients undergoing curative surgical resection for oesophagogastric cancer.

## Methods

This systematic review is reported in accordance with PRISMA^16^ and MOOSE^17^ guidelines. The protocol was prospectively registered on PROSPERO (CRD42021293019)^18^ following a search to ensure no prior review had been performed on this topic. Ethical approval was not required for this work.

### Search strategy, inclusion and exclusion criteria

A systematic search of MEDLINE (PubMed), EMBASE (OVID) and Cochrane Library databases, from January 2000, was conducted on 24 May 2022. Details of the search strategy are outlined in *Appendix S1*. Search results were synthesized and managed using web-based review software ‘Covidence’ (Veritas Health Innovations, Melbourne, Australia) and duplicates were removed. Titles and abstracts of identified articles were independently screened by two reviewers (L.R.B./J.S.) for potentially relevant studies. Those selected were subject to full text review. To identify further relevant studies, the reference lists of included articles were searched manually. In instances of discrepancy regarding inclusion, consensus was met by consultation between reviewers or with a senior author (R.J.E.S.).

Inclusion criteria were studies that examined adult patients (≥18 years) undergoing surgical resection for cancer of the oesophagus or stomach with curative intent and studies that recorded the presence of cachexia, or involuntary weight loss, prior to neoadjuvant or surgical treatment. Inclusion was irrespective of subsequent use of adjuvant therapies. If the reported patient cohort included patients treated with palliative intent, further information was sought from the authors, by email, regarding the outcomes of the non-palliative cohort only. Those that were unable to provide outcome data for only the curatively resected patients were ineligible for inclusion or subsequent analysis. In cases where studies originated from the same centre or included data from the same database, then only the more recent publication or largest cohort was included. Exclusion criteria were: conference abstracts, systematic reviews, or case reports (*n* < 5 patients), studies that included patients who had surgery with palliative intent, where the authors were unable to provide data for the non-palliative subgroup, studies that did not include overall survival as an outcome measure, studies published before the year 2000 and studies not published in the English language.

### Definitions of cachexia

Cachexia was defined as per any of the previously published objective diagnostic criteria. In short, Evans *et al*. defined cachexia as BMI < 20 kg/m^2^ or weight loss > 5 per cent (over the preceding 12 months) alongside three of five other features: fatigue, decreased muscle strength, anorexia, low fat-free mass index or abnormal biochemistry^8^. In 2009, Bozzetti and Mariani defined cachexia as ≥10 per cent loss from a stable pre-morbid weight^9^. Most recently, the Fearon *et al*. definition required >5 per cent weight loss or >2 per cent if BMI < 20 kg/m^2^ for a diagnosis of cachexia^6^.

### Data extraction and outcomes of interest

Data were independently extracted from included studies by two authors (L.R.B./J.S.) using an extraction template developed in advance of the literature search. This included year of publication, study location, study type, data collection period, patient demographics, disease details and weight loss or cachexia criterion used. Study authors were approached by email regarding missing data. The primary outcome of interest was overall survival (1-year, 3-year and 5-year survival). Secondary outcomes included disease-free survival and postoperative mortality.

### Risk of bias assessments

Risk of bias assessment was performed independently by two researchers (L.R.B./M.Y.) using the ROBINS-E tool for non-randomized studies of exposure^19^. Following individual grading, each study was discussed between reviewers to ensure consensus. In instances of any disagreement, a third senior author was consulted as required (R.J.E.S.). Funnel plots were constructed to assess publication bias.

### Statistical analysis

The meta-analysis was conducted in accordance with Cochrane Collaboration recommendations^20^. Study characteristics, patient demographics and disease details were reported descriptively. Where possible, outcome data were extracted directly from the included manuscripts. This was primarily the adjusted HR, with 95 per cent c.i., demonstrating the influence of preoperative cachexia on overall survival. Studies which provided a Kaplan–Meier plot were digitized using WebPlotDigitizer^21^ to calculate survival proportions for each time-point of interest. Meta-analyses of proportions were then conducted to calculate pooled 1-, 3- and 5-year survival. Heterogeneity was assessed using *I*^2^ analysis and publication bias was assessed using a funnel plot. Random effects modelling, using the DerSimonian and Laird method, was utilized throughout. Statistical significance was defined as *P* ≤ 0.050. Meta-regression was performed to examine the potential for heterogeneity as a result of variation in disease stage between studies. All statistical analyses were performed using R 4.2.2 (R Foundation for Statistical Computing, Vienna, Austria) with packages including *meta* and *metafor*.

## Results

**Fig. 1:**
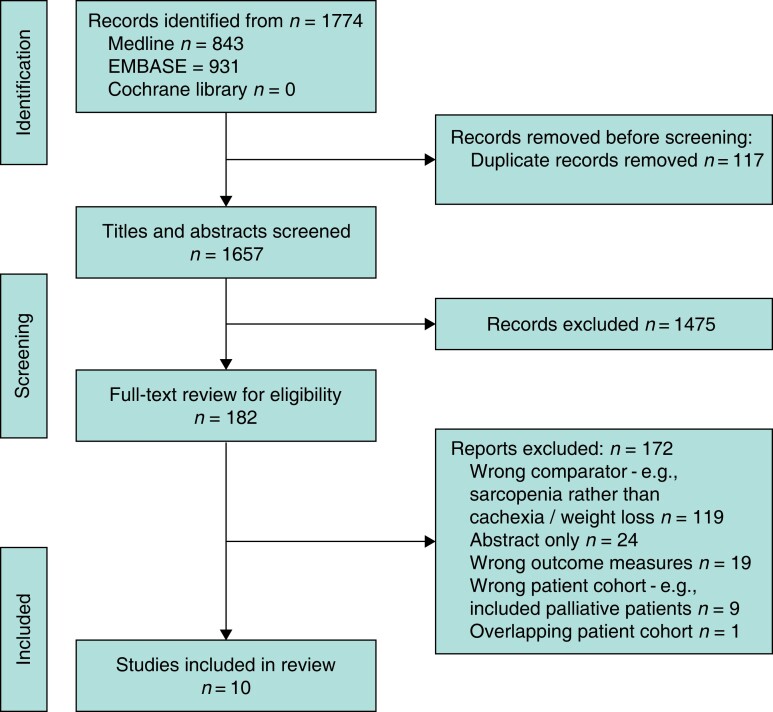
PRISMA flow chart

A total of 1774 studies were identified through initial searches of Medline, EMBASE and Cochrane Library databases. After removal of duplicates (*n* = 117), the title and abstract of 1657 unique studies were screened for inclusion. Of these, 1474 articles were deemed irrelevant and 182 studies were retrieved for full-text review. Following detailed screening against inclusion and exclusion criteria, 10 studies were eligible for quantitative synthesis (*Fig. 1*).

### Study characteristics and risk of bias

**Fig. 2:**
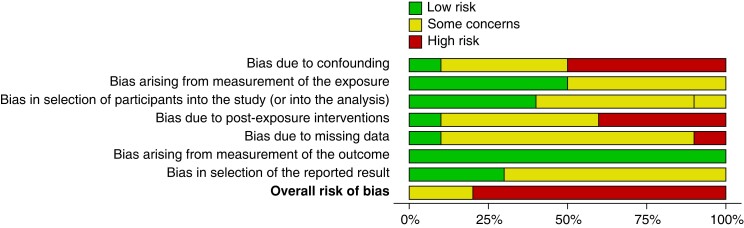
Risk of bias (ROBINS-E) summary plot

All included studies were observational cohort studies, either prospective (50 per cent)^22–26^ or retrospective (50 per cent)^27–31^ in nature (*Table 1*). The majority of studies were from Asia (60 per cent)^24,25,28–31^ with the remainder undertaken in Europe^22,23,26,27^. Risk of bias was deemed moderate/high for all 10 studies (*Fig. 2* and *Appendix S2*)^32^.

**Table 1:** Characteristics of included studies

Authors	Year of publication	Study type	No. of centres	Study country	Inclusion period
Skipworth *et al.*^22^	2009	PCS	Multi-centre	United Kingdom	January 2001–March 2004
Van der Schaaf *et al.*^27^	2014	RCS	Single centre	The Netherlands	May 1990–October 2010
Liu *et al.*^28^	2016	RCS	Single centre	China	January 2003–December 2012
Hynes *et al.*^23^	2017	PCS	Multi-centre	Sweden	April 2001–December 2005
Guner *et al.*^29^	2018	RCS	Single centre	South Korea	March 2009–December 2015
Yu *et al.*^30^	2018	RCS	Single-centre	China	January 2012–December 2013
Chen *et al.*^24^	2019	PCS	Multi-centre	China	January 2014–December 2016
Zhang *et al.*^31^	2020	RCS	Single centre	China	August 2005–December 2011
Dijksterhuis *et al.*^26^	2021	PCS	Multi-centre	The Netherlands	January 2015–December 2018
Zhuang *et al.*^25^	2022	PCS	Single centre	China	July 2014–March 2019

RCS, retrospective cohort study; PCS, prospective cohort study.

### Patient characteristics

A total of 7186 patients who underwent resection of OG cancer were included in quantitative analyses. Most larger patient cohorts were reported by studies based in Asia (*Table 1*)^24,25,28–31^. Median age varied between 59 and 67 years, and a consistent male preponderance was seen, with proportions ranging from 61 to 83 per cent (*Table 2*). While the distributions of BMI at diagnosis were variable, European cohorts had a notably higher number of overweight/obese patients. Among the five studies that reported co-morbidity, many patients (32–58 per cent) had no pre-existing diagnoses.

**Table 2:** Patient characteristics

Authors	Sample	Age (Years)	Sex (% Male)	BMI (kg/m^2^)	Comorbidity (%)	Cachexia (%)	Weight loss comparator
Skipworth *et al.*^22^	93	Mean 67	61%	<25 (42%) >25 (58%)	Not reported	48%	>10% weight loss (pre-morbid stable)
Van der Schaaf *et al.*^27^	922	Mean 63 (s.d.: 10)	77%	<25 (45%) 25–29 (31%) >30 (10%)	None (46%) ≥1 Comorbidity (54%)	17%	>10% weight loss (3 months prior)
Liu *et al.*^28^	1026	Median 59 (range: 19–89)	68%	<18.5 (39%) 18.5–24.9 (34%) >25 (27%)	Not reported	32%	>5% weight loss (6 months prior)
Hynes *et al.*^23^	390	Mean 66	81%	<25 (51%) 25–30 (32%) >30 (17%)	CCI: 0 (58%) CCI: 1 (24%) CCI: ≥2 (18%)	24%	>10% weight loss (pre-morbid stable)
Guner *et al.*^29^	1032	Median 60 (i.q.r.: 9)	65%	Median 23 (i.q.r.: 21–26)	None (53%) ≥1 co-morbidity (47%)	Not reported	>5% weight loss (3 months prior)
Yu *et al.*^30^	121	<55 (46%) ≥55 (54%)	81%	Median 22 (range: 15–29)	Not reported	55%	>5% weight loss (6 months prior)
Chen *et al.*^24^	575	Mean 64 (s.d.: 11)	75%	Median 22 (i.q.r. 20–24)	CCI: 0 (51%) CCI: 1–4 (45%) CCI: ≥4 (4%)	36%	Fearon *et al.* cachexia definition*
Zhang *et al.*^31^	1545	Mean: 60 (s.d.: 8)	83%	<18.5 (14%) 18.5–24.9 (74%) >25 (12%)	Not reported	36%	>8.8% weight loss (3 months prior)
Dijksterhuis *et al.*^26^	267	Mean 64.4 (s.d. 8.2)	77%	Median 25.7 (i.q.r. 23.5–28.6)	None (32%) ≥1 co-morbidity (53.0%) Unknown (15.0%)	45%	Fearon *et al.* cachexia definition*
Zhuang *et al.*^25^	1215	Median 65 (i.q.r.: 14)	73%	Mean 22 (s.d.: 3)	CCI: 0–1 (91%) CCI: ≥2 (9%)	27%	Fearon *et al.* cachexia definition*

CCI, Charlson Comorbidity Index^33^; i.q.r., interquartile range. *Fearon *et al*. defined cachexia as >5 per cent weight loss or >2 per cent weight loss with BMI<20 or sarcopenia (6 months prior).

### Disease characteristics and treatment details

Included studies considered patients with oesophageal (*n* = 2978) and gastric (*n* = 3848) cancer (*Table 3*). Two studies reported on a mixed OG cohort (*n* = 360). Most patients with oesophageal disease from Asian cohorts had squamous cell carcinoma whereas more adenocarcinoma of the oesophagus was observed in European populations. Cancer stage varied considerably between studies, with rates of stage III/IV disease ranging between 17 and 58 per cent.

**Table 3:** Disease characteristics and treatment details

Authors	Tumour site	Histological subtype	Clinical stage	Neoadjuvant/adjuvant therapies	Operative details
Skipworth *et al.*^22^	Oesophageal and gastric	Not reported	Stage III/IV (54%)	Not reported	Oesophagectomy (44%) Total gastrectomy (10%)
Van der Schaaf *et al.*^27^	Oesophageal	AC (61%) SCC (39%)	Stage III/IV (48%)	NA RTx and/or CTx (22%)	Trans-hiatal oesophagectomy (79%) Trans-thoracic oesophagectomy (18%) Other (3%)
Liu *et al.*^28^	Gastric	Well differentiated (17%) Poorly differentiated (83%)	Stage III (58%)	Adjuvant CTx (62%)	Not reported
Hynes *et al.*^23^	Oesophageal	AC (76%) SCC (24%)	Stage III/IV (49%)	NA CTx (6%)	Oesophagectomy (80%) Extended total gastrectomy (10%) Oesophago-gastrectomy (10%)
Guner *et al.*^29^	Gastric	Not reported	Stage III (17%)	Not reported	Total gastrectomy (21%) Subtotal gastrectomy (79%)
Yu *et al.*^30^	Oesophageal	SCC (100%)	T3/4 (78%) N1/2/3 (60%)	Adjuvant CTx (100%)	Trans-thoracic oesophagectomy (100%)
Chen *et al.*^24^	Gastric	Differentiated (73%) Undifferentiated (9%) Signet ring cell (18%)	Stage III (46%)	Not reported	Subtotal gastrectomy (100%)
Zhang *et al.*^31^	Oesophageal	SCC (100%)	T3/4 (67%) N1/2/3 (42%)	NA treatment (0%) Adjuvant treatment (37%)	Sweet oesophagectomy (81%) Ivor–Lewis oesophagectomy (9%) McKeown oesophagectomy (9%)
Dijksterhuis *et al.*^26^	Oesophageal and gastric	AC (75%) SCC (24%) Other (1%)	Stage III/IV (55%)	Not reported	Not reported
Zhuang *et al.*^25^	Gastric	Not reported	Stage III (39%)	Not reported	Not reported

AC, adenocarcinoma; SCC, squamous cell carcinoma; NA, neoadjuvant; RTx, radiotherapy; CTx: chemotherapy.

The use and nature of anti-cancer therapies was poorly reported, with neoadjuvant and adjuvant treatments being described by only one study^27^ and three studies, respectively^28,30,31^. The types of surgical resection undertaken were described more frequently^22–24,27,29–31^. Among those studies that reported operative approach, the majority performed all resections as open surgery^22,27,30,31^. Only Guner *et al*. reported a minimally invasive approach in 76 per cent of their patients who underwent gastrectomy^29^.

### Comparison between cachexia and weight stable disease

The percentage cut-offs and time frames used to define cachectic and weight-stable groups varied between studies. However, all were within the ranges of those used in the contemporary published definitions of cachexia^6,8,9^. As such, all weight-losing participants met the diagnostic criteria for cachexia. In the studies that compared patients based on a diagnosis of cachexia^24–26^, all used the Fearon *et al.* definition^6^. All but one study^29^ reported the proportion of patients undergoing an OG resection who were weight-losing or cachectic. Across the nine remaining studies (*n* = 6270), the pooled prevalence of pre-treatment cachexia was 35 per cent (95 per cent c.i.: 24–47 per cent).

### Primary outcome: overall survival

**Fig. 3:**
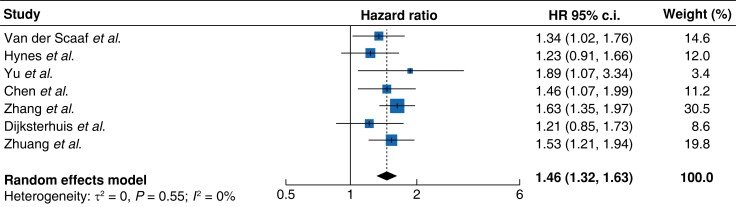
Pooled adjusted hazard ratios for the effect of cachexia on overall survival

The influence of pre-treatment cachexia on overall survival of patients following resection of an OG cancer was reported by nine studies. Of these, seven presented an adjusted hazard ratio^23–27,30,31^ while two presented only a univariable analysis^28,29^. Meta-analysis of the adjusted hazard ratios confirmed that cachexia was associated with a decreased overall survival (HR 1.46 (95 per cent c.i.: 1.32–1.65), *P* < 0.001; *Fig. 3*). Heterogeneity between the included studies was low (*I*^2^ = 0 per cent) and visual inspection of the funnel plot suggested no evidence of publication bias (*Appendix S3*). Analysis of unadjusted hazard ratios estimated a similarly adverse effect of pre-treatment cachexia (HR 1.76 (95 per cent c.i.: 1.28–2.41); *Appendix S4*).

Subgroup analysis by tumour site confirmed cachexia as an adverse prognostic marker for both oesophageal (HR 1.47 (95 per cent c.i.: 1.26–1.72); *Appendix S5a*) and gastric cancer (HR 1.51 (95 per cent c.i.: 1.25–1.82); *Appendix S5b*). The negative influence of cachexia was also evident on subanalysis of European cohorts (HR 1.27 (95 per cent c.i.: 1.07–1.51); *Appendix S6a*), with an even greater adverse effect seen in Asian studies (HR 1.58 (95 per cent c.i.: 1.39–1.80); *Appendix S6b*). Data were not available to allow subgroup analysis by histological subtype of oesophageal cancer.

### Primary outcome: overall survival at 1, 3 and 5 years

Survival at both 1- and 3-years was reported by six studies (*n* = 3196)^22,24–27,30^. Pooled survival at 1 year was 79 per cent (95 per cent c.i.: 74–83 per cent) among patients with cachexia and 88 per cent (95 per cent c.i.: 82–92 per cent) in those who were weight stable (*Appendix S7a, b*). Lower rates of survival in cachectic patients were similarly evident at 3 years (48 per cent (95 per cent c.i.: 38–58 per cent) *versus* 67 per cent (95 per cent c.i.: 55–76 per cent); *Appendix S8a, b*). Survival at 5 years was reported by three studies (*n* = 2404)^25–27^. A pooled 5-year survival of only 41 per cent (95 per cent c.i.: 25–60 per cent) was estimated among cachectic cohorts, compared with 57 per cent (95 per cent c.i.: 37–74 per cent) in those who maintained their weight prior to treatment (*Appendix S9a, b*).

### Secondary outcomes: disease-free survival and postoperative mortality

Three studies reported the influence of pre-treatment cachexia on disease-free survival following OG cancer resection^23,25,29^. Meta-analysis revealed that this was not an adverse predictor of disease-free survival (HR 1.23 (95 per cent c.i.: 0.98–1.55), *P* = 0.077; *Appendix S10*).

Only Skipworth *et al*. (cachectic: 2 per cent *versus* weight stable: 6 per cent) and Van der Schaaf *et al.* (cachectic: 10 per cent *versus* weight stable: 7 per cent) compared rates of postoperative mortality between cachectic and weight stable groups. Owing to this paucity of data, no quantitative synthesis was performed.

### Pre-treatment cachexia in the context of disease stage

**Fig. 4:**
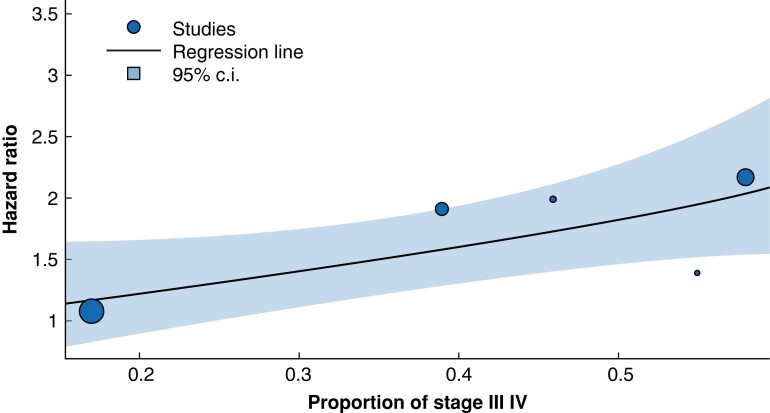
The influence of disease stage on cachexia’s effect on survival

The proportion of patients with clinical stage III/IV disease was reported by five of the studies^24–26,28,29^ that provided an unadjusted hazard ratio for the effect of pre-treatment cachexia on survival. The proportion of patients with locally advanced disease was found to be a significant moderator of between-study heterogeneity (*R*^2^ = 58 per cent, *P* = 0.027). The model's estimated regression weight was 1.38 (95 per cent c.i.: 0.15–2.60) suggesting a greater effect on overall survival is evident in studies with a higher proportion of stage III/IV disease (*Fig. 4*).

## Discussion

Pre-treatment cachexia is adversely associated with overall survival following surgical resection for an oesophageal or gastric cancer. This may be particularly evident for patients with a more advanced disease stage. Data regarding the secondary outcome measures of disease-free survival and postoperative mortality were more limited and further investigations in this area are required. Consideration of cachexia as a negative prognostic marker may allow modification of treatment pathways for higher-risk patients. Alternatively, targeted multi-modal intervention, with pre- or rehabilitative measures, may help ameliorate the effects of cachexia in surgical cohorts.

A challenge with the study of weight loss in patients with OG cancer is the complex interplay between the pathophysiological mechanisms that underpin it. Alongside the local obstructive effects of the tumour, a large proportion of patients with OG cancer will develop cancer cachexia^5^. Unlike those who simply have mechanically impaired intake, cachectic patients won't recover with nutritional supplementation alone^6^. The pooled cachexia prevalence of 35 per cent, calculated in the present review, was only slightly lower than estimates from the existing literature (∼40–65 per cent) that consider both curative and non-curative cancer stages^5,33–35^. While variation was evident in the weight loss cut-offs used, all were clearly within the ranges of those used in the published definitions of cachexia. However, inconsistencies in definitions or patient descriptors can often result in treatment effect heterogeneity and consensus on diagnostic criteria would benefit future cachexia research.

All three included studies that used cachexia, rather than weight loss, as a comparator^24–26^, based this on Fearon *et al.*'s diagnostic criteria^6^. The absence of factors such as inflammatory markers, anaemia and fatigue from this definition has previously been a source of criticism. It has been suggested that the focus on weight loss, when not considered alongside other phenotypical features, could lead to an over-diagnosis of cachexia, and that Evans *et al.*'s definition^8^ is prognostically superior for overall survival^36^. However, the complexity of diagnostic criteria in the Evans *et al.* definition has likely contributed to its decreased utilisation. The 2018 Global Leadership Initiative on Malnutrition (GLIM) criteria^37^ highlighted the need to consider systemic inflammation, as a key driver of cachexia. Very few of the included studies described pre-treatment inflammatory markers despite their known prognostic value in OG cancer cohorts^38^.

The adverse impact of radiologically evident sarcopenia on survival has been demonstrated in gastric^11^ and oesophageal cancers^12^. Only one of the included studies reported the presence of sarcopenia alongside cachexia^25^. Following adjustment for confounders, their findings suggested that, while both were prognostic, cachexia had a greater influence on survival. Sarcopenia is highly prevalent across both curative and non-curative OG cancer^14^. The low specificity of this marker presents difficulty with its clinical application and highlights an inability to differentiate between new dynamic wasting and pre-morbid sarcopenia. Poor baseline muscularity will usually be associated with comorbidity, age and frailty. While both will likely be prognostic, the effect of the primary sarcopenic phenotype will differ from that of cancer-related wasting.

While weight loss is the cardinal feature of cachexia, other surrogate markers can, and should, be considered alongside it. The cachexia grading system, using weight-loss adjusted for BMI, was devised across a mixed (palliative and curative) cancer cohort^39^ and found to be prognostic by one of the included studies^31^. The application of this grading alongside other cachectic markers (performance status and modified Glasgow Prognostic Score (mGPS)) has shown further risk-stratification utility in advanced cancer^40^. BMI must be duly considered when diagnosing cachexia, as the presence of obesity is known to mask weight loss as a presenting symptom^39^. It could be postulated that the prevalence of cachexia may be understated in higher BMI cohorts, and members of our group have shown previously that sarcopenic obesity to be a high-risk nutritional syndrome in upper gastrointestinal cancer^41^. Conversely, the so-called ‘obesity paradox’ (observed improved survival rates in overweight patients) is also a recognized phenomenon in cancer care^42^. Across the included studies, the proportion of overweight patients varied considerably, particularly between European and Asian cohorts. These differences, alongside other factors, such as histological subtype, may account for some of the observed variation on geographical subgroup analysis. The authors of the present review hypothesise that the combination of sarcopenia with other features of cachexia (for example, weight loss, biochemical markers of muscle wasting^43^ or inflammation) could help differentiate between static pre-morbid and disease-related poor muscularity. Future studies could even work towards the development of a more detailed scoring system, synthesizing clinical, biochemical^44^ and radiological markers, to more robustly ‘stage the host’.

A limitation of this review was the paucity of reported detail regarding the use of (neo) adjuvant anti-cancer therapies. These treatments can drive muscle wasting and may worsen cancer-related symptoms such as dysphagia or anorexia, thus promoting further weight loss. As such, this remains a potential source of between-study heterogeneity. A number of papers have previously assessed tissue wasting during neoadjuvant therapies and have shown an adverse effect on overall survival^45^. Unlike low muscle mass or density on a single baseline staging scan, this type of assessment provides evidence of ongoing dynamic wasting, albeit confounded by the use of neoadjuvant anti-cancer therapies. Future research would benefit from longitudinal radiological assessment, thus characterizing temporal trends in wasting.

The proportion of patients with advanced stage disease varied considerably (17–78 per cent) across the included studies. As such, this confounder was examined as a potential source of heterogeneity using meta-regression. The results suggest that pre-treatment cachexia may have a greater influence on overall survival in studies with a higher proportion of advanced stage OG cancer. Only Lui *et al*. performed subgroup analyses regarding this, and their findings similarly suggested cachexia was less prognostic in early stage disease^28^. Further patient-level evaluation, with adjustment for confounders, is required. Patients with stage III/IV cancers represent a more ‘borderline’ resectable population, where treatment selection is less clear and postoperative survival is often limited. As such, the prognostic effect of weight loss could be of particular relevance to this group. Some of these patients may even have, unidentified (micro)metastatic disease, which could represent a potential driver of cachexia^46^. It could be postulated that the pathophysiology underlying weight loss in more advanced malignancy may differ from that of earlier stage disease. Examination of weight loss alongside other markers may help clarify whether cachexia or poor dietary intake represent the predominant driving force behind tissue loss in more advanced disease. Cachexia, by definition, cannot be reversed by increased nutritional intake alone^6^. It is likely that multi-modal therapies, utilizing dietary supplementation, exercise and medical therapies (for example, anti-inflammatories or immune checkpoint inhibitors), are required for the treatment of this multi-factorial syndrome. Future prehabilitation trials could target this cachectic yet operable cohort.

This is the first review to highlight the importance of pre-treatment cachexia as a prognostic marker in patients planned for curative resection of an oesophageal or gastric cancer. Consideration of this, during the shared decision-making process, offers the potential for better risk stratification, targeted intervention and ultimately improved outcomes.

## Supplementary Material

znad239_Supplementary_Data

## Data Availability

Data are available from the corresponding author upon reasonable request.
