# Supplementary material for: The prognostic impact of pre-treatment cachexia in resectional surgery for oesophagogastric cancer: a meta-analysis and meta-regression
Source: Br J Surg. 2023 Aug 1;110(12):1703–11. doi: 10.1093/bjs/znad239 (PMC10638534; doi:10.1093/bjs/znad239)
Supplement: znad239_Supplementary_Data [file znad239_supplementary_data.zip › Supplementary_Material.docx]

***Appendix 1: Search Strategy***

*Medline (PubMed):*

(("Cachexia" [MeSH] OR "Sarcopenia" [MeSH] OR “Weight Loss” [MeSH] OR “Body Mass Index” [MeSH]) AND ("Esophageal Neoplasms" [MeSH] OR “(o)esophageal cancer” OR "Stomach Neoplasms" [MeSH] OR “gastric cancer” OR “upper gastrointestinal cancer” OR “upper GI cancer”) AND ("surg*")).

*EMBASE (OVID):*

((exp Cachexia/ OR exp Sarcopenia/ OR exp Body Weight Loss/ OR exp Body Mass/) AND (exp Esophagus Cancer/ OR exp Stomach Cancer/) AND (exp Cancer Surgery))

***Appendix 2: Risk of Bias (ROBINS-E) Summary*
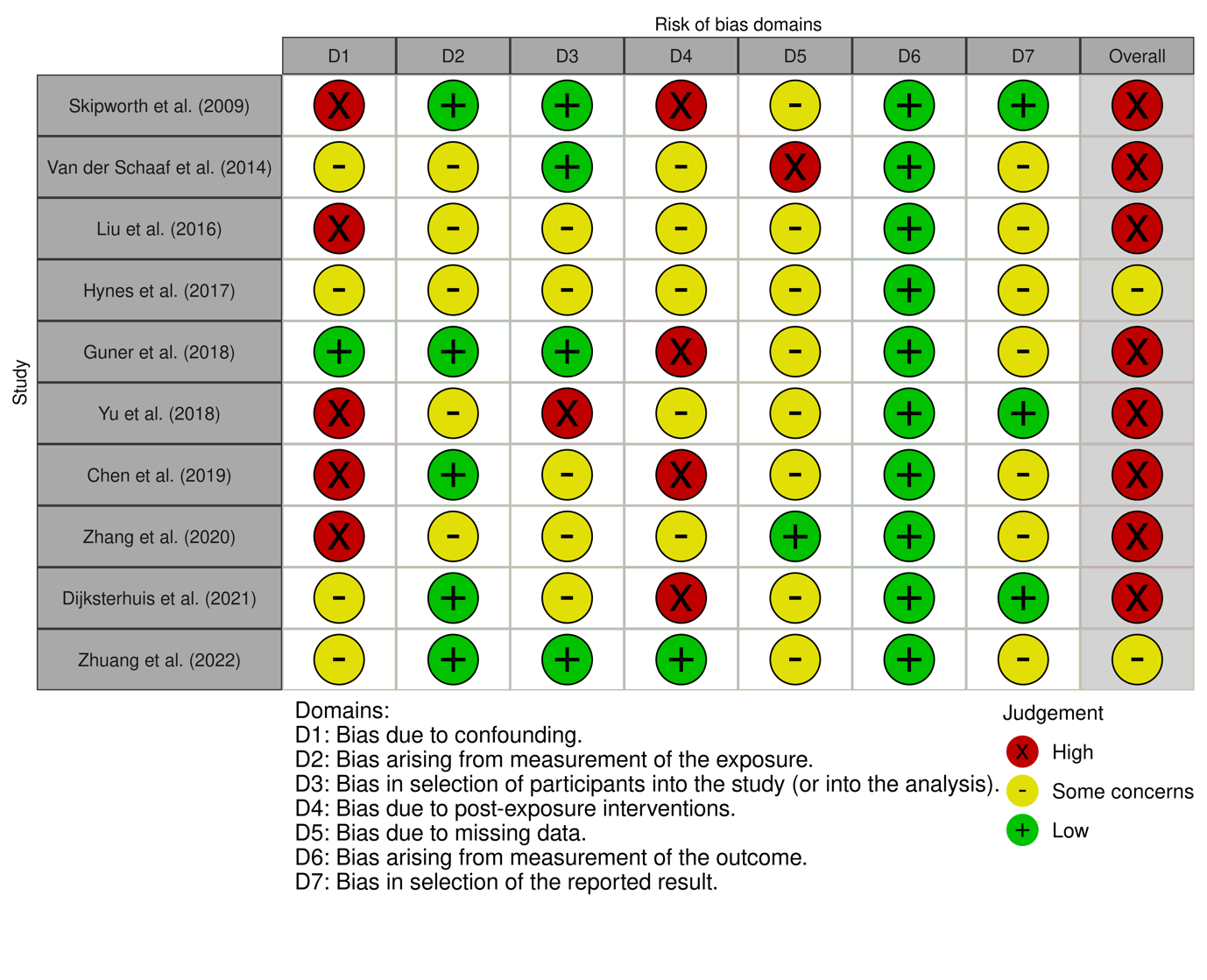
**

***Appendix 3: Funnel Plot of Adjusted Hazard Ratio for Influence of Cachexia on Survival***

***
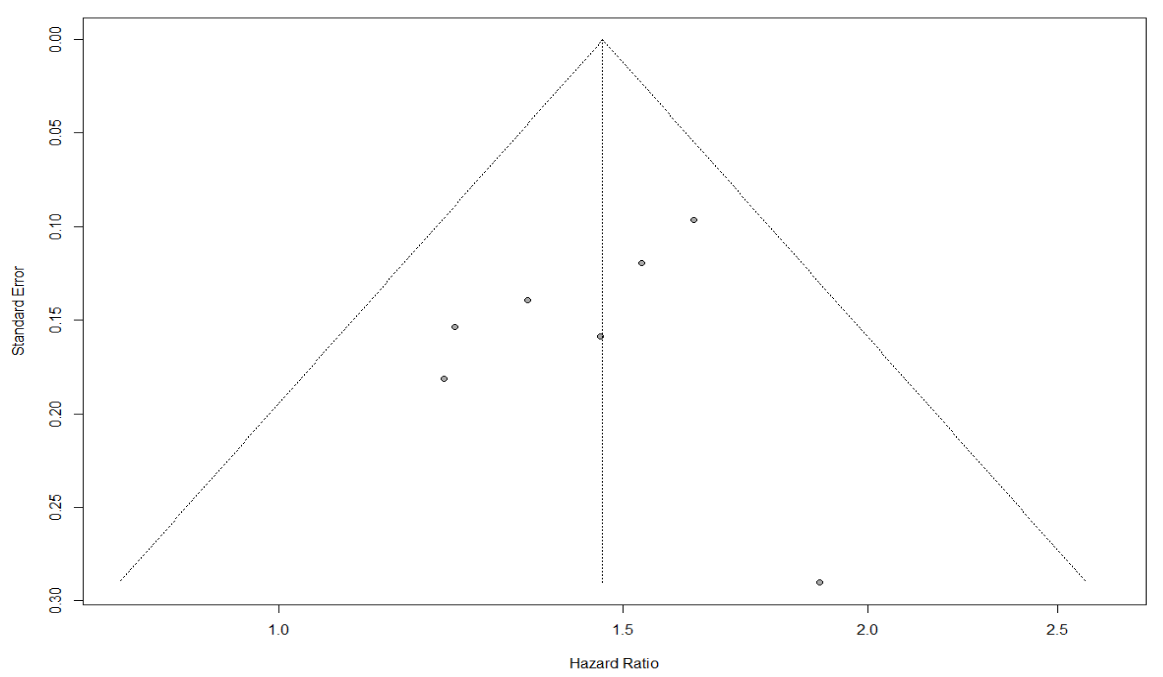
***

***Appendix 4: Pooled Unadjusted (Univariable) Hazard Ratios for the Effect of Cachexia on Overall Survival***

***
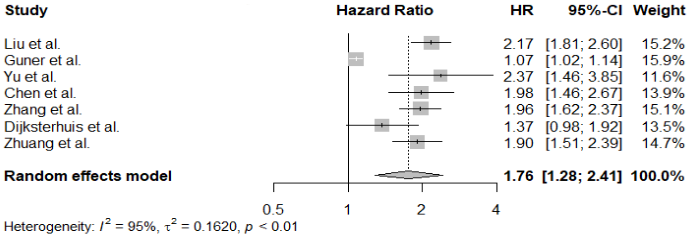
***

***Appendix 5a: Pooled Adjusted Hazard Ratios for the Effect of Cachexia on Overall Survival in Oesophageal Cancer***

***
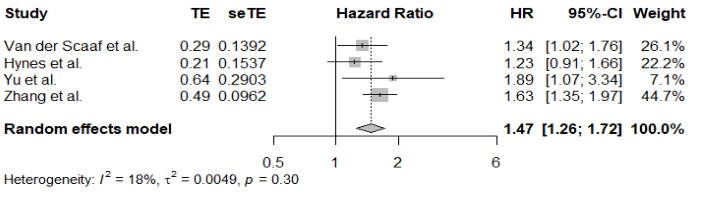
***

***Appendix 5b: Pooled Adjusted Hazard Ratios for the Effect of Cachexia on Overall Survival in Gastric Cancer***

***
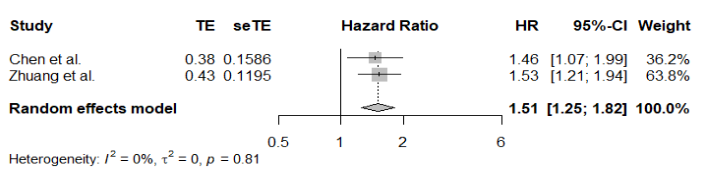
***

***Appendix 6a: Pooled Adjusted Hazard Ratios for the Effect of Cachexia on Overall Survival in European Cohorts***

***
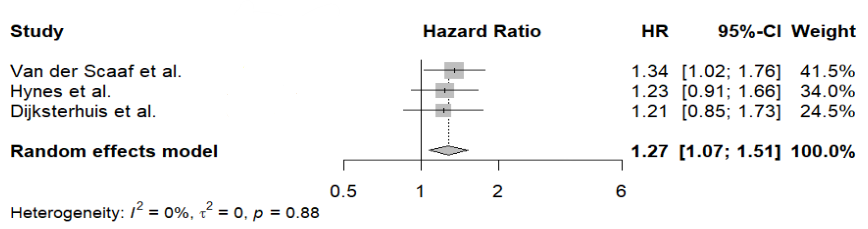
***

***Appendix 6b: Pooled Adjusted Hazard Ratios for the Effect of Cachexia on Overall Survival in Asian Cohorts***

***
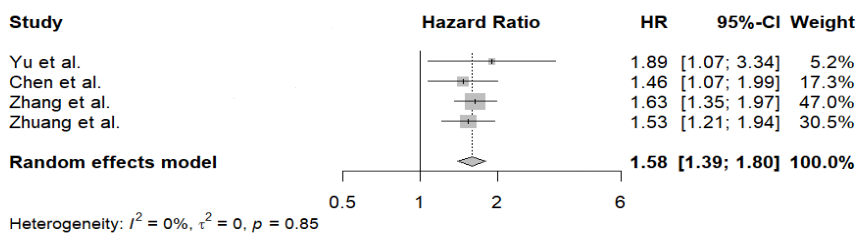
***

***Appendix 7a: Pooled Survival at 1 Year in Weight Stable Patients***

***
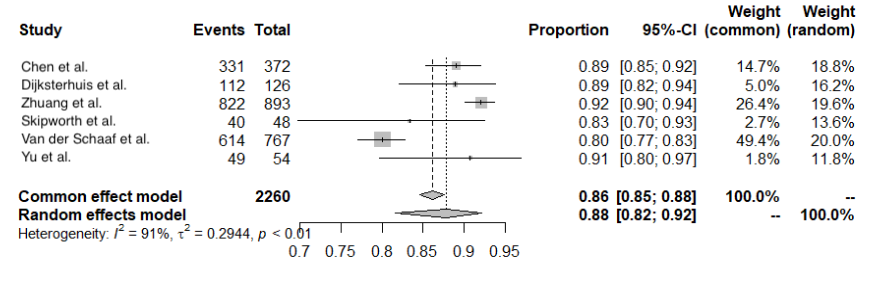
***

***Appendix 7b: Pooled Survival at 1 Year in Cachectic Patients***


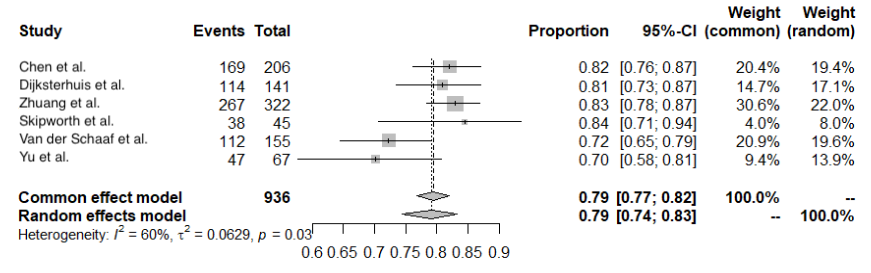


***Appendix 8a: Pooled Survival at 3 Years in Weight Stable Patients***

***
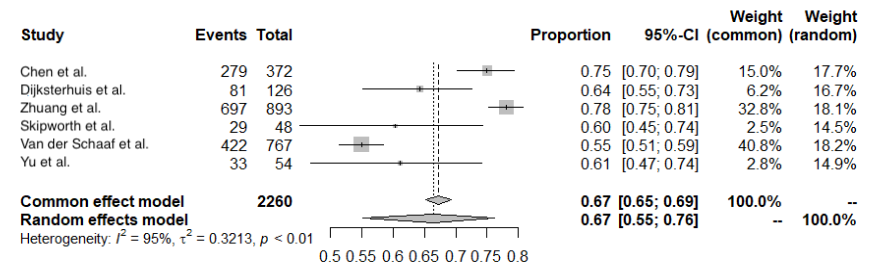
***

***Appendix 8b: Pooled Survival at 3 Years in Cachectic Patients***

***
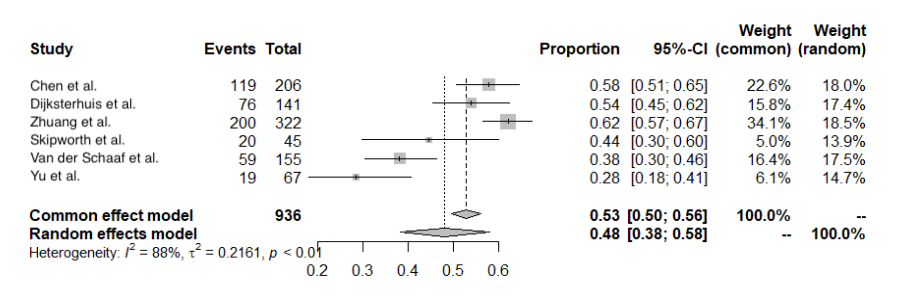
***

***Appendix 9a: Pooled Survival at 5 Years in Weight Stable Patients***

***
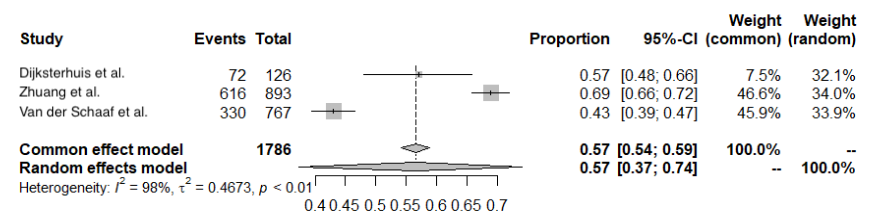
***

***Appendix 9b: Pooled Survival at 5 Years in Cachectic Patients***

***
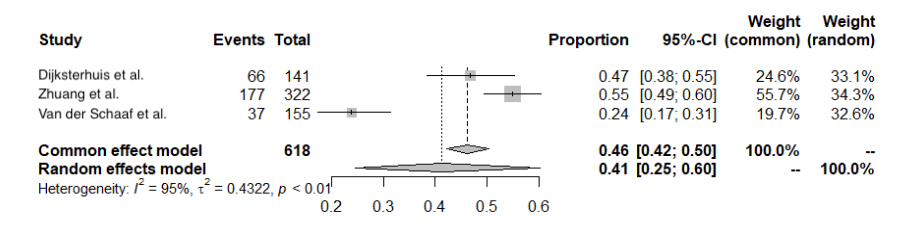
***

***Appendix 10: Forest Plot of Studies Reporting the Impact of Cachexia on Disease-Free Survival***

***
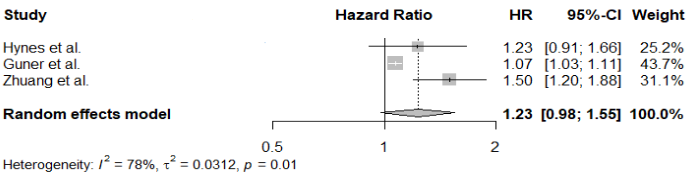
***
